# Partitioned Memory Storage Inspired Few-Shot Class-Incremental Learning

**DOI:** 10.3390/e28080843

**Published:** 2026-07-29

**Authors:** Yimin Yin, Wanxia Deng, Jing Zhang, Xiayan Cheng, Jinghua Zhang

**Affiliations:** 1 School of Mathematics and Statistics, Hunan First Normal University, Changsha 410205, China; yinyimin1@126.com; 2 Hunan Provincial University Key Laboratory for Big Data Analysis and Application, Hunan First Normal University, Changsha 410205, China; 3 College of Meteorology and Oceanography, National University of Defense Technology, Changsha 410073, China; wanxiadeng@163.com; 4 Key Laboratory of Computing and Stochastic Mathematics, Hunan Normal University, Changsha 410081, China; zhangjing_nudt@163.com; 5 College of Computer Science and Software Engineering, Hohai University, Nanjing 211100, China

**Keywords:** deep learning, few-shot learning, incremental learning, uncertainty quantification, information entropy

## Abstract

Current mainstream deep learning techniques exhibit an over-reliance on extensive training data and a lack of adaptability to the dynamic world, marking a considerable disparity from human intelligence. To bridge this gap, Few-Shot Class-Incremental Learning(FSCIL) has emerged, focusing on continuous learning of new categories with limited samples without forgetting old knowledge. Existing FSCIL studies typically use a single model to learn knowledge across all sessions, inevitably leading to the stability–plasticity dilemma. Unlike machines that usually consolidate all categories into a single parameter space, cortical memory organization suggests that different types of knowledge can be distributed and organized across specialized cortical regions. Inspired by this organization principle, our paper aims to develop a method that learns independent models for each session. It can inherently prevent catastrophic forgetting. During the testing stage, our method integrates Uncertainty Quantification (UQ) for model deployment. Our method provides a fresh viewpoint for FSCIL and demonstrates the state-of-the-art performance on CIFAR-100 and mini-ImageNet datasets.

## 1. Introduction

**Figure 1:**
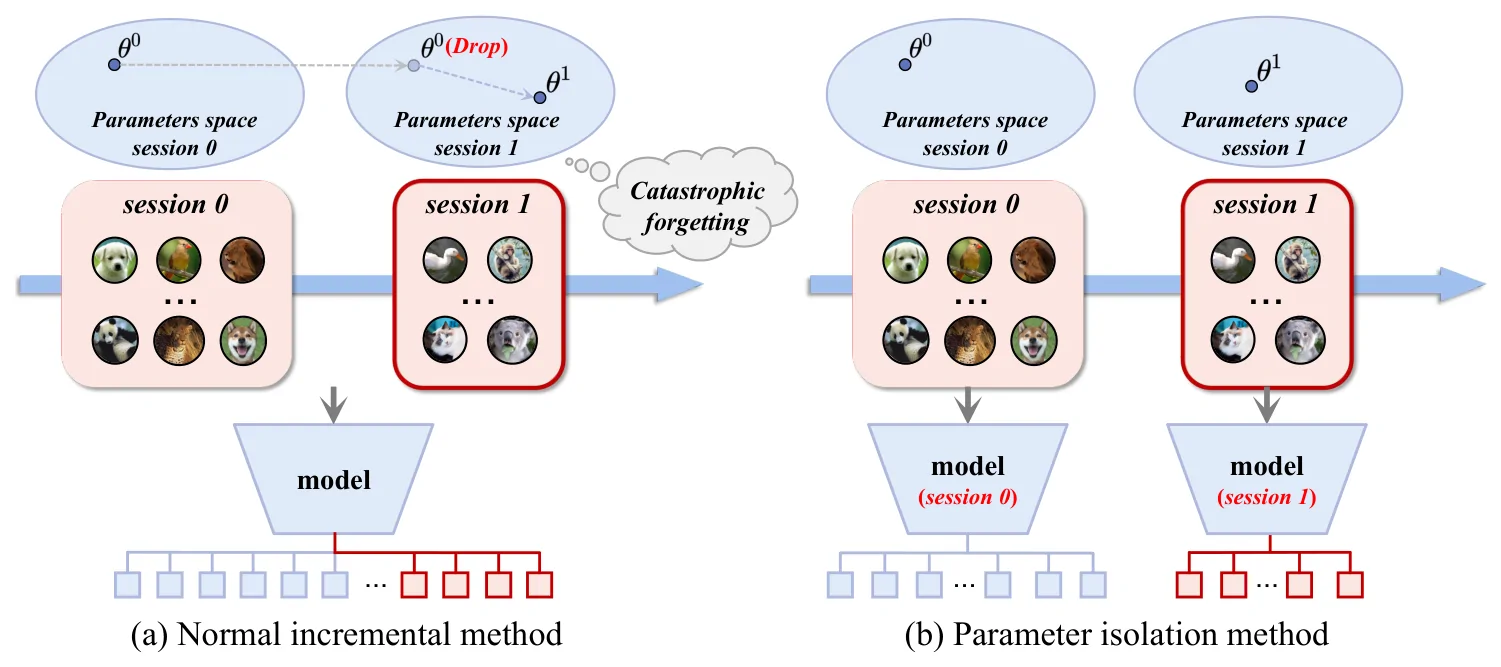
Comparing ordinary incremental learning method to parameter-isolation method. $\theta^{t}$ represents the model parameter on session *t*. (**a**) Model parameters change, triggering catastrophic forgetting. (**b**) The model for each session learns the data independently and saves the parameters separately.

Deep learning has achieved significant milestones in numerous large-scale computer vision tasks [1,2,3]. These approaches generally involve learning a mapping from samples to corresponding labels using extensive datasets. However, the mapping learned from a specific data distribution is usually fixed and non-extensible, i.e., a model can only recognize trained categories and cannot generalize to new categories. To enable the trained model to generalize effectively to new categories, Class Incremental Learning (CIL) has received widespread attention [4,5]. CIL strives to enable models to continually learn different categories from data streams instead of a fixed dataset, while preserving the capability to recognize previously encountered categories. Most CIL studies focus on letting models learn incrementally when the new class samples are sufficient. However, acquiring samples from new categories proves challenging and resource-intensive in many practical scenarios [6]. To tackle the challenge posed by the scarcity of samples from new categories, a more challenging and practically significant task, denoted as FSCIL, has emerged. In contrast to CIL, FSCIL must incrementally learn in situations with extremely limited labeled samples for new categories. Current FSCIL research predominantly adheres to the traditional CIL paradigm, wherein a single model assimilates all data throughout the incremental process, sharing identical model parameters and decision boundaries. This learning paradigm presents significant challenges in conserving the model’s old memory and deviates from cortical memory organization. As shown in Figure 1, acquiring knowledge of new categories inevitably alters parameters trained on old ones, causing catastrophic forgetting.

In contrast to this memory-burdened and forgettable learning paradigm, cortical memory organization provides an important inspiration: acquired knowledge can be distributed across specialized cortical regions while remaining accessible for later retrieval [7,8]. Such organization motivates a learning system that stores session-specific knowledge separately while still supporting global recognition. However, from the standpoint of computational modeling, the challenges faced by deep neural networks in drawing inspiration from cortical memory organization within FSCIL are principally manifested in the following two aspects. (i) How can the model imitate the organized and distributed storage of acquired knowledge? The unavailability of session information underscores the necessity for the model to possess the capability to recognize all previously learned categories. This limitation results in the necessity for the model to store the knowledge acquired across all sessions entirely within a unified memory area that cannot be segregated. (ii) How can one establish a mapping from task to memory? The session from which the test sample comes is unknown in FSCIL, so establishing a mapping relationship between the samples and the model becomes unfeasible, i.e., the session to which the test sample belongs is unknown. This implies that directly mapping a test sample to the relevant memory component, as suggested by cortical memory organization, is not feasible. This paper is dedicated to bridging the gap between the learning process of FSCIL and cortical memory organization by addressing the aforementioned challenges.

In this paper, to tackle the first challenge of enabling the classification system to store memories in an organized and session-specific manner, inspired by cortical memory organization, we adopt a parameter-isolation method, training distinct classification models for each session. In the conventional FSCIL, the knowledge pertaining to all sessions is consolidated within a singular model. This consolidation poses a risk of catastrophic forgetting. In our parameter-isolation method for FSCIL, each model exclusively stores knowledge acquired in its respective session, and the decision boundaries of these models operate independently, as shown in Figure 1b. This approach to retaining previous knowledge effectively mirrors the distributed organization principle of cortical memory. Furthermore, to mitigate the overfitting of new categories, we use the branch training strategy to train a series of models. To address the second challenge of establishing a mapping from samples to models, we predict the session information of the samples by UQ. Test samples are input into each model individually to obtain classification results along with corresponding uncertainty values. Afterward, the appropriate classification result is selected based on the uncertainty value. UQ enables the model not only to produce its classification results but also to express the level of uncertainty associated with a given sample. The model gives low uncertainty to categories it recognizes and gives high uncertainty to categories it has never encountered. Relying on UQ, we successfully construct a model-to-memory mapping inspired by cortical memory organization. The model preserving the corresponding session memory is identified from a range of models for each test sample.

To validate the effectiveness of the proposed method, we conduct comprehensive comparative and ablation experiments on multiple benchmark datasets. The contribution of this work is summarized as follows:

- We propose a fresh viewpoint for FSCIL, inspired by cortical memory organization. Our work is the first parameter-isolation method in FSCIL. Our approach achieves state-of-the-art performance on benchmark datasets.
- In the testing phase where session information is not accessible, we employ information entropy to quantify the uncertainty of a sample, enabling the inference of session information about the sample.
- We employ a novel data augmentation strategy for real categories to generate virtual prototypes. This strategy significantly enhances the feature extraction capability of the backbone.

## 2. Related Work

### 2.1. Few-Shot Learning

FSL aims to recognize unseen objects from a few labeled samples [9]. Currently, FSL research can be mainly categorized into metric-based methods [10,11,12,13,14], optimization-based methods [15,16,17,18], and model-based methods [19,20,21]. The metric-based approaches use the algorithm of nearest neighbor to learn the relationship between pairs of samples and assign labels to the samples with the smallest distance by calculating the distance between pairs of samples. Munkhdalai and Yu [20] use the mean of all samples in each category to represent the position of each category in the embedding space. In our approach, features are extracted using a pre-trained backbone and then classified by computing cosine similarity [11] between the samples and the prototypes in the embedding space.

### 2.2. Class-Incremental Learning

In the domain of class-incremental learning, the model consistently learns new knowledge from a data stream characterized by a diverse array of sample categories. This paper focuses on a class-incremental learning challenge that emerges when confronted with a constrained number of samples. iCaRL [22] utilizes the nearest neighbor classifier to learn new categories and employs knowledge distillation as a strategy to preserve the knowledge of existing categories. LUCIR [23] enhances performance in subsequent sessions by searching flat minima during the base training process. Ref. [24] proposes the same hypothesis to LUCIR: the base model plays a critical role in the overall performance of the incremental task. It establishes a robust feature extractor through training on base categories, subsequently freezing it. Then, for each incremental session, it fine-tunes the tail layers of the feature extractor and classifier individually. Inspired by [24], in this paper, we employ the strategy of fine-tuning the tail of the feature extractor in FSCIL for incremental learning. Compared to fully freezing the feature extractor, this strategy allows the model to better adapt to new categories.

### 2.3. Few-Shot Class-Incremental Learning

FSCIL represents a more challenging learning task compared to class-incremental learning. FSCIL aims to continuously acquire knowledge pertaining to new categories with a limited amount of labeled data while simultaneously preventing the inadvertent forgetting of previously learned categories [25]. The problem setting of FSCIL was first proposed in TOPIC [26]. It utilizes the Neural Gas (NG) network to stabilize the topological manifold features between new categories and old categories. CEC [25] uses a graph neural network to update the classifier parameters in each incremental session based on the global knowledge of previous sessions. Simultaneously, it creates pseudo-incremental scenarios to optimize the graph neural network during base training. The pseudo-incremental approach has been used in many studies [6,27]. Unlike previous approaches that only consider the performance of the current session, [6] uses a method that enhances the scalability of the incremental model by compacting the embedding space through the inversion of virtual prototypes. Beyond these, several other representative methods have been proposed to tackle FSCIL from different perspectives. F2M [28] argues that the base model plays a decisive role in the incremental process and improves the performance of subsequent sessions by searching for flat minima during base training. MetaFSCIL [29] adopts a meta-learning framework that samples pseudo-incremental episodes during base training, enabling the model to learn how to incrementally adapt to new categories while alleviating forgetting. Entropy-reg [30] performs entropy-regularized data-free replay, in which synthesized samples are used to preserve old knowledge without retaining the original training data. GKEAL [31] formulates the incremental process as an analytic learning problem and employs a Gaussian kernel embedding together with a recursive least-squares solution, allowing new categories to be incorporated in a closed-form manner without gradient-based fine-tuning. In this paper, we crop and mix images with different categories to form images of virtual categories, relying on semantic information similar to that of real categories to generate virtual prototypes.

### 2.4. Parameter-Isolation Methods in Continual Learning

Parameter-isolation methods address catastrophic forgetting by allocating separate model parameters for different tasks, thereby preventing interference between old and new knowledge. Progressive Neural Networks [32] pioneered this direction by instantiating a new network column for each task and connecting it to previous columns via lateral connections, ensuring that previously learned parameters remain strictly frozen. PackNet [33] takes a complementary approach by iteratively pruning and recycling redundant parameters, assigning dedicated parameter subsets to each task without expanding the network. More recently, DER [34] proposes a dynamically expandable representation that incrementally augments the feature extractor with new network branches for each task while freezing earlier ones, achieving strong performance in class-incremental learning. Building upon this, FOSTER [35] further introduces a feature compression stage to counteract the model expansion caused by continual branch addition. In the domain of dynamic architecture methods, DyTox [36] leverages a Vision Transformer backbone and introduces dedicated task tokens for each incremental task, enabling task-conditioned parameter specialization. More recently, parameter-isolation ideas have been extended to prompt-based and adapter-based designs. EASE [37] constructs task-specific expandable subspaces via lightweight adapters for each incremental task, allowing new knowledge to be encoded without overwriting previously learned representations. MOS [38] further mitigates parameter drift across tasks by merging task-specific adapters, striking an improved balance between knowledge isolation and inter-task consolidation.

Despite their effectiveness, these methods typically rely on task identity at test time to select the corresponding parameter subset, whereas session labels are unavailable during FSCIL inference. They are also not designed for the few-shot constraint in incremental sessions. In contrast, our method introduces parameter isolation into FSCIL and uses entropy-based uncertainty quantification to select the best-matching session model without access to session labels.

## 3. Problem Setting

The fundamental aim of FSCIL is to perpetually learn novel categories from a few labeled samples, all the while safeguarding the retention of previously acquired knowledge. In FSCIL, the complete training dataset $D_{train}$ can be represented as $\left\{D_{train}^{\left(0\right)},D_{train}^{\left(1\right)},D_{train}^{\left(2\right)},\ldots ,D_{train}^{\left(N\right)}\right\}$, where $D_{train}^{\left(t\right)}$ represents the training data of the *t*-th session, and *N* is the total number of sessions. The corresponding label space is $\left\{C_{train}^{\left(0\right)},\ldots ,C_{train}^{\left(N\right)}\right\}$. The samples and their labels from different sessions do not overlap, ∀i,j∈{0,1,…,N} and i≠j, $D_{train}^{\left(i\right)}\cap D_{train}^{\left(j\right)}=⌀$ and $C_{train}^{\left(i\right)}\cap C_{train}^{\left(j\right)}=⌀$. $D_{train}^{\left(0\right)}$ is the base training dataset, which contains a large number of categories where each category has abundant training samples. $D_{train}^{\left(i\right)}\left(i\in \left\{1,\ldots ,N\right\}\right)$ is the incremental training dataset, which contains only a few samples; it contains *N* categories, and each category contains *K* samples, known as *N*-way *K*-shot. During the training in the *t*-th session, the model only interacts with the samples and labels from the current session, $D_{train}^{\left(i\right)}$ and $C_{train}^{\left(i\right)}$. During the testing phase of the *i*-th session, the evaluation involves all the categories that the model has already learned $\left\{C_{train}^{\left(0\right)}\cup C_{train}^{\left(1\right)}\cup \ldots \cup C_{train}^{\left(i\right)}\right\}$.

## 4. Methodology

**Figure 2:**
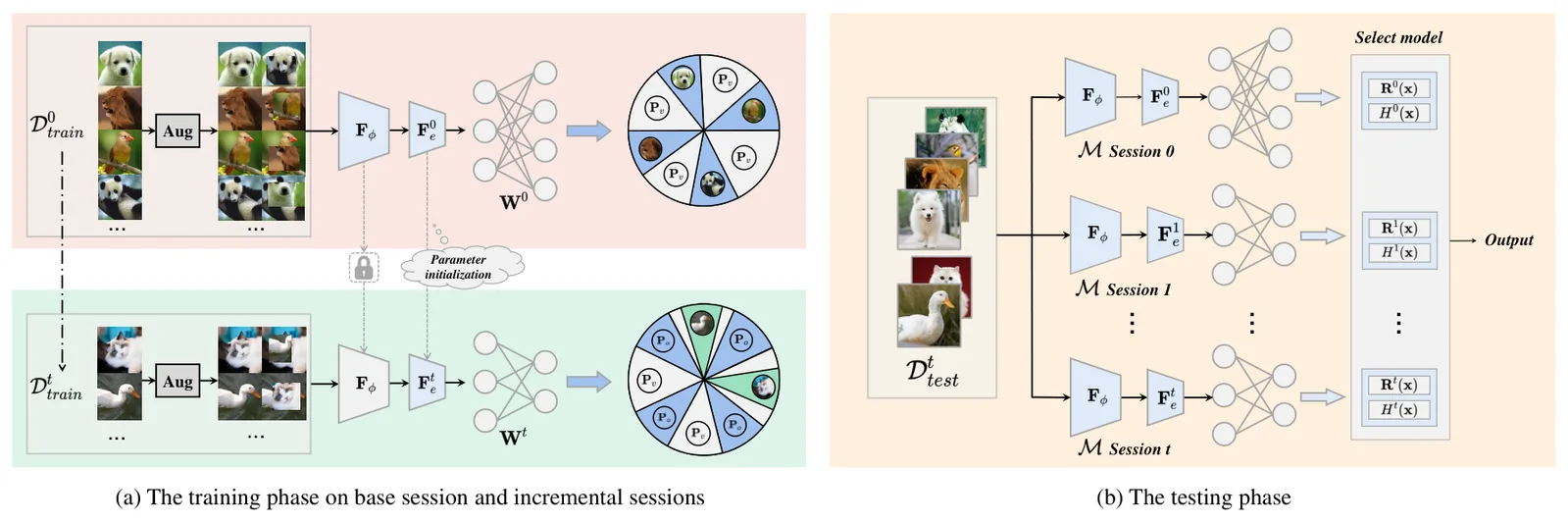
The overview of our proposed cortical-memory-organization-inspired FSCIL approach. (**a**) The CutMix data augmentation method enhances the feature extractor’s performance by generating virtual prototypes. For session *t*, freeze the bulk $F_{\phi }$ of the feature extractor, fine-tune the tail $F_{e}^{t}$ and the classifier $W^{t}$. (**b**) In the testing stage, the samples x are fed to a trained series of models outputting category probabilities $R^{t}\left(x\right)$ and uncertainty $H^{t}\left(x\right)$. The reliable result is then selected based on the value of $H^{t}\left(x\right)$.

In this paper, we utilize a forward-compatible framework based on virtual prototype generation to enhance the backbone. It simulates incremental scenarios during the training phase of the base session to provide a prospective view of the model. In the incremental stage, we train the entire model using a representation learning decoupling strategy and a parameter isolation mechanism between sessions, saving parameters separately for each session. This is the first parameter-isolation method in FSCIL. In the testing stage, we use UQ based on information entropy to select the best-matching classification result for the sample. The overall framework of our proposed method is shown in Figure 2.

### 4.1. Forward-Compatible Framework

**Figure 3:**
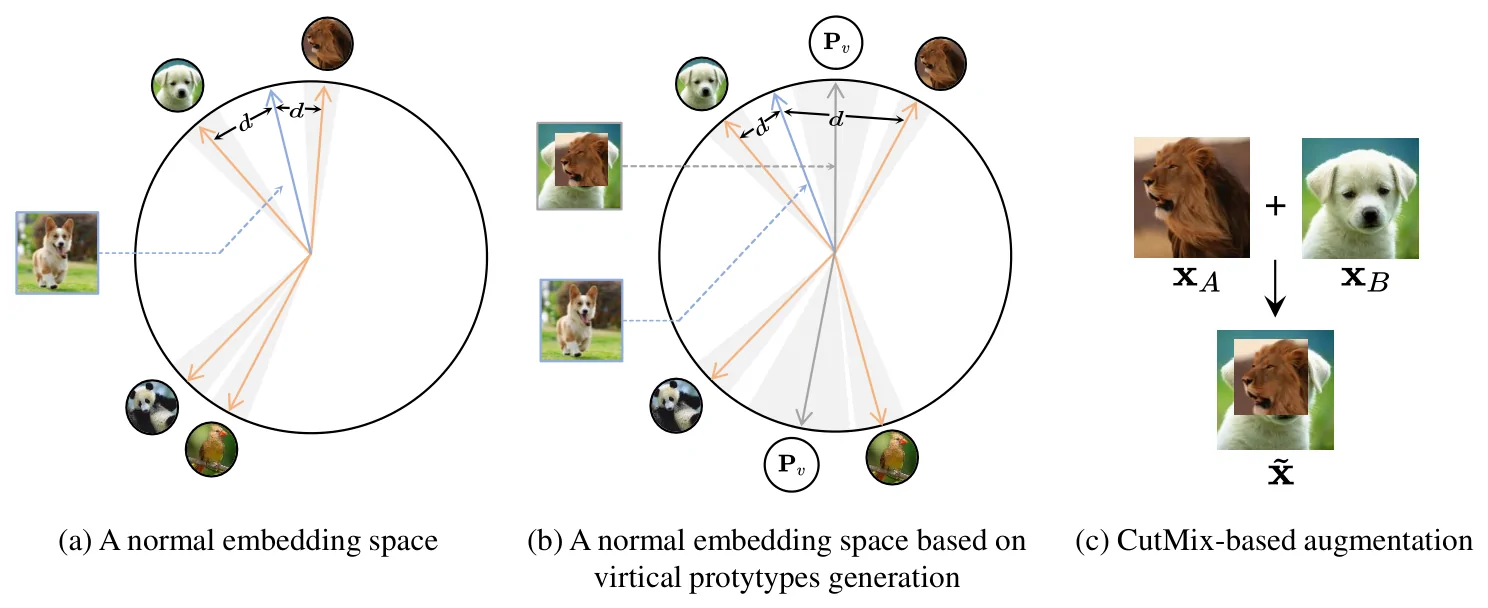
Virtual prototypes in embedding space. (**a**) Samples determine their category based on the closest prototype to them. Samples are more likely to be misclassified when the prototypes are too close to each other. (**b**) Inserting the virtual prototypes increases the separation between real prototypes and the embedding space’s sparsity. (**c**) Procedure for virtual sample generation.

FSCIL typically employs sufficient samples to optimize the empirical loss of the training data, thereby ensuring the robustness of the backbone [6]. Nevertheless, this approach can only learn decision boundaries for existing categories, making it incompatible between the new and old sessions. The incompatibility precludes the continuity between sessions, resulting in the insertion of new prototypes that disrupt the structure of the old session’s embedding space. To facilitate the coordination of prototype distribution across the data stream as a whole rather than within the confines of individual sessions, we employ a forward-compatible framework based on virtual prototype generation. It utilizes virtual categories to simulate incremental scenarios during the training phase of the base session, allowing the embedding space to be reserved for future occurrences of the category, giving a forward-looking view of the entire model, and ensuring compatibility between different sessions. Our forward-compatible framework uses CutMix [39] to generate images of virtual categories. It takes an image that has been cropped and scaled and overlays it on an image from another category, as detailed in Figure 3. Equation (1) shows the process of cropping and scaling image $x_{B}$ onto image $x_{A}$.

(1) $$\tilde{x}=M⊙x_{A}+\left(1-M\right)⊙x_{B},\;M\in {\left\{0,1\right\}}^{H\times W}$$

Here, $\tilde{x}$ denotes the image with the virtual category from the fusion of $x_{A}$ and $x_{B}$, M is a binary mask that drops image information, and ⊙ represents pixel-by-pixel multiplication. We restrict M to half the original image size to integrate semantic features from both categories. The virtual samples generated by CutMix align closely with real categories in the embedding space. This facilitates the recognition of samples by the distance metric, as the spacing between real prototypes is increased. Furthermore, the compression of existing prototypes allocates sufficient space for new categories. After training a sufficiently powerful feature extractor, samples from the real category are used to fine-tune the classifier to make the target category consistent with the real category.

### 4.2. Decoupling in Representation Learning for Parameter-Isolation

**Figure 4:**
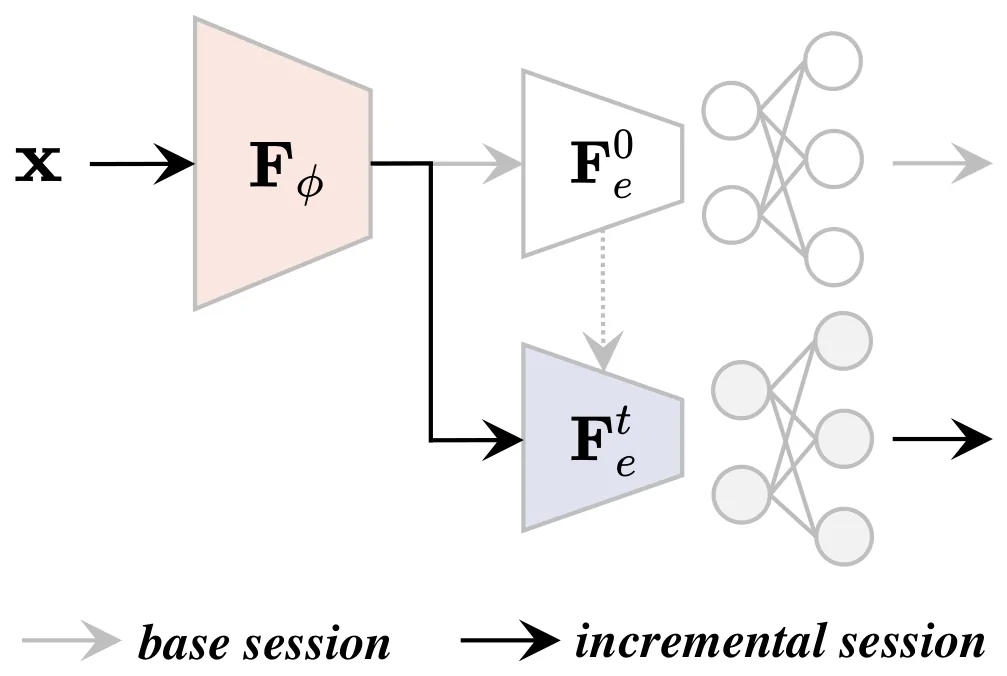
Decoupled training strategy used in this paper. x represents the inputs. During incremental training, the majority of the feature extractor $F_{\phi }$ is frozen to suppress catastrophic forgetting, while the tails $F_{e}^{t}$ and classifiers undergo fine-tuning.

Previous FSCIL strategies decouple the feature extractor used to learn the representation and the classifier during the incremental process, freezing the parameters of the feature extractor to preserve the old class knowledge [25,30]. However, the decoupling of representation learning and the classifier obliterates the model’s ability to learn representations of new categories. In fact, for a strong pre-trained feature extractor, fine-tuning its tails with new data can make it perform better on new sessions [24]. To facilitate the acquisition of more category-specific features during incremental sessions and the formation of more precise decision boundaries, we decouple the representation learning process, rather than merely decoupling the classifiers. Specifically, we partition the feature extractor into two parts, the main component $F_{\phi }$ and the tail layer $F_{e}$. With sufficient data in the base session, we train $F_{\phi }$, $F_{e}^{0}$, and the classifier $W^{0}$. Due to the scarcity of samples in the incremental process of FSCIL, fine-tuning the whole model can fall into an overfitting dilemma. Therefore, in incremental sessions, the parameters of $F_{\phi }^{t}$ are frozen to preserve the fundamental representational capabilities, and the $F_{e}^{t}$ and $W^{t}$(t≥1) undergo fine-tuning to learn the representation of new categories. Figure 4 illustrates this decoupled training strategy. Our decoupling strategy for representation learning permits the model to learn features about new categories during each incremental session, rather than extracting feature vectors using parameters from the base session. To allow decoupled models to avoid catastrophic forgetting while learning new categories, we use a parameter isolation strategy to keep separate model parameters for each session. The parameters of $F_{\phi }$ are shared for each session, and $F_{e}^{t}$ and $W^{t}$ are stored separately for each session.

The feature vector $p_{i}$ extracted from the image $x_{i}$ by the feature extractor is shown in Equation (2). The feature extractor extracts all training samples of class *k* to generate feature vectors and computes the mean to obtain the feature prototype $\bar{p^{k}}$ for that class. The feature prototypes are calculated as shown in Equation (3). In the base session and incremental sessions, the cross-entropy loss function as shown in Equation (4) is used for training.

(2) $$p_{i}=F_{e}\left(F_{\phi }\left(x_{i}\right)\right)$$

(3) $$\bar{p^{k}}=\frac{1}{\left|C(k)\right|}\sum_{i=1}^{\left|C(k)\right|}p_{i}^{k}$$

(4) $$L_{CE}=\sum_{j=1}^{N}\log\left(\frac{e^{\cos<\bar{p^{k}},p_{j}^{k}>}}{\sum_{i=1}^{\left|C\right|}e^{\cos<\bar{p^{i}},p_{j}^{k}>}}\right)$$

### 4.3. Model Selection Based on Uncertainty Quantification

During FSCIL testing, without access to session labels, mapping samples to the correct model poses a critical challenge. During the testing stage, we have a feature extractor backbone $F_{\phi }$, a sequence of feature extractor tails $\left\{F_{e}^{0},F_{e}^{1},\ldots ,F_{e}^{n}\right\}$ and a sequence of classifiers $\left\{W^{0},W^{1},\ldots ,W^{n}\right\}$. Choosing the right one from a range of models for a test sample is critical. UQ can measure the model’s certainty level with the input samples, producing uncertainty values for classification. In this study, we use information entropy to measure the uncertainty of the input samples. The information entropy is calculated as shown in Equation (5).

(5) $$H^{t}\left(x\right)=-\sum_{c=1}^{|C^{t}|}p\left(o^{c}\right)\log p\left(o^{c}\right)$$

Here, $H^{t}\left(x\right)$ denotes the information entropy of the model in session *t* for the fed sample x, $|C^{t}|$ denotes the total number of categories in session *t*, and $p(o^{c})$ represents the probability of class *c* for the input sample x. During the testing stage, the samples are fed into all session models. Simultaneously, each model computes the uncertainty of the input samples based on Equation (5). $p(o^{c})$ is obtained by mapping the neural network output to a probability. Finally, the model with the minimum uncertainty is chosen as the classification model for the given sample, and the predicted class from that model is accepted.

#### Category Imbalance in Uncertainty Quantification

Identical target categories for both classification systems are crucial to render the results of the UQ meaningful and comparable for analysis. However, in FSCIL’s setting, base sessions comprise many categories, while incremental sessions feature fewer. To maintain consistency in target categories between the base session model and incremental session models during UQ, we partition the probabilities generated by the base session model into $N_{sub}$ sub-results, where $N_{sub}$ is shown in Equation (6).

(6) $$N_{sub}=\frac{|C^{0}|}{|C^{i}|},\;\left|C^{i}\right|=\left|C^{j}\right|\left(1\leq i,j\leq n\right)$$

$|C^{0}|$ and $|C^{i}|$ represent the number of categories in the base session and the incremental session, respectively. The output of the base session model has a total of $N_{sub}$ sub-results, where each sub-result contains the probability of $|C^{i}|$ categories. We quantify the uncertainty for each sub-result and select the smallest as the UQ result for the whole base session model. The uncertainty values obtained before the sub-result division are shown in Equation (7). The uncertainty values obtained after dividing the results of the base session classification are shown in Equation (8).

(7) $$U=\{H^{0}\left(x_{i}\right),H^{1}\left(x_{i}\right),\ldots ,H^{n}\left(x_{i}\right)\}$$

(8) $$U=\{H_{1}^{0}\left(x_{i}\right),\ldots ,H_{N_{sub}}^{0}\left(x_{i}\right),H^{1}\left(x_{i}\right),\ldots ,H^{n}\left(x_{i}\right)\}$$

Here, $U_{\mathrm{base}}=\left\{H_{1}^{0}\left(x_{i}\right),\ldots ,H_{N_{sub}}^{0}\left(x_{i}\right)\right\}$ denotes the set of all sub-results from the base session model. The minimum value in $U_{\mathrm{base}}$ is used as the uncertainty value of the base session model and participates in the UQ containing all session models. This sub-result partitioning strategy for the base session model effectively tackles the category imbalance between the base and incremental categories. This is achieved by aligning the categories in the sub-results of the base session with the incremental classes.

## 5. Experiments

### 5.1. Dataset

CIFAR-100 contains 100 classes with 600 32×32 RGB images per class, of which 500 are used for training and 100 for testing, and the entire dataset has 600,000 images. A total of 60 classes are used as base classes, 40 as incremental classes, and eight sessions are in the incremental stage. The data in each incremental session are organized as 5-way 5-shot. mini-ImageNet is a subset of ImageNet that contains 100 classes, each containing 600 84×84 RGB images. A total of 60 classes are used as base classes and 40 are incremental classes. The 40 classes are evenly distributed into eight sessions, with five classes per session. The data in each incremental session are organized as 5-way 5-shot.

### 5.2. Implementation Details

Following other FSCIL studies [25,30,31], we employ ResNet-18 as the backbone to validate the performance of our proposed method on the benchmark datasets. We follow the setting of [25,31] for CIFAR-100 and mini-ImageNet. For the training/test splits, we consistently follow the standard settings adopted by the FSCIL community [26] to ensure fair performance comparisons. We use randomly initialized model parameters. The entire feature extraction section of ResNet-18 is partitioned into four blocks, each consisting of three convolution layers. The first three blocks are $F_{\phi }$, where the parameters are frozen after training in the base session. The last block in the tail is $F_{e}$, and it continuously updates the parameters during incremental sessions. For the detailed training configuration, the feature extraction backbone in the base-session training stage is trained for 300 epochs, and the classifier fine-tuning stage in the base session is conducted for 200 epochs. In each incremental session, the model is fine-tuned for 15 epochs. The learning rates for the base session and incremental sessions are set to 0.1 and 0.175, respectively, and stochastic gradient descent (SGD) is adopted as the optimizer. All programs are run on a single NVIDIA GeForce RTX 4070 Laptop GPU. The Python and PyTorch versions are 3.9 and 2.1, respectively.

### 5.3. Comparison with the State-of-the-Art Methods

**Figure 5:**
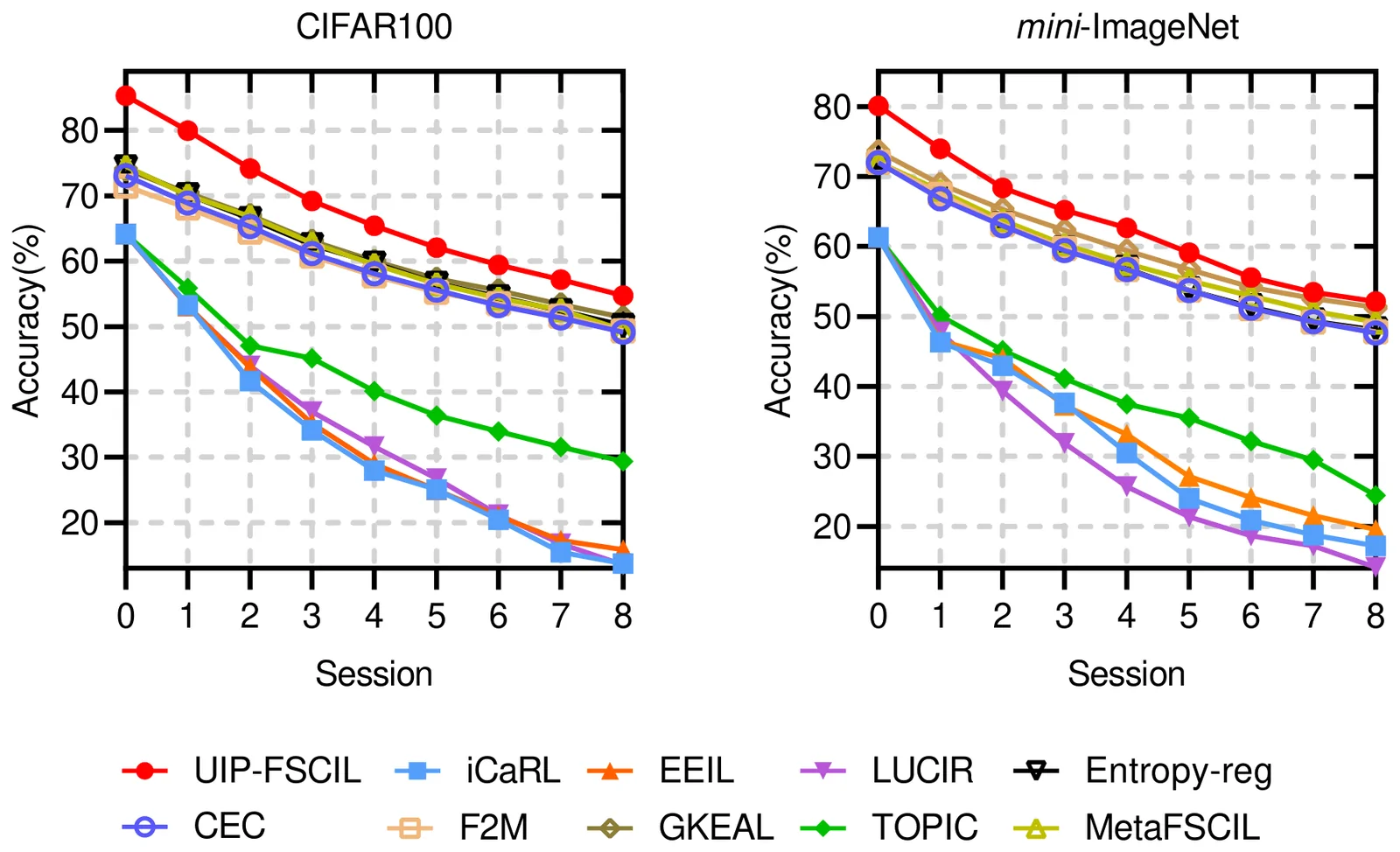
Average accuracy in each session of two benchmark datasets: CIFAR-100 and mini-ImageNet.

To validate the state-of-the-art performance of our method, we conduct comparative experiments with contemporary approaches on two benchmark datasets: CIFAR-100 and mini-ImageNet. The comparative performance of our method compared to other FSCIL methods is shown in Figure 5. Furthermore, the detailed performance for CIFAR-100 is shown in Table 1. We compare only with methods using comparable problem settings, backbone families, and training protocols to ensure fair evaluation. Accordingly, foundation-model-based methods are not included because their large-scale pretraining and resource requirements differ substantially from the standard FSCIL setting.

Based on the experimental results, our approach attains satisfactory performance for both CIFAR-100 and mini-ImageNet, demonstrating excellent performance in both the base session and incremental sessions. In CIFAR-100, our method outperforms GKEAL [31] by 3.73% in the final session, with an average accuracy improvement of 6.09%. The experimental results unequivocally demonstrate that our method outperforms other state-of-the-art methods for FSCIL datasets. Our proposed parameter-isolation approach in FSCIL effectively mitigates the issue of catastrophic forgetting. Simultaneously, the experimental results substantiate that the UQ technique can construct a mapping from samples to models. This learning method offers a fresh perspective for the FSCIL field.

### 5.4. Parameter Analysis

In addition to classification accuracy, the number of model parameters is an important factor for evaluating the practicality of FSCIL methods. Since the proposed method maintains session-specific components while sharing the major feature extraction backbone, we report the parameter scale of the model across different sessions to provide a clearer view of its storage cost and scalability. The detailed parameter statistics are summarized in Table 2.

As shown in Table 2, the shared backbone remains unchanged across all sessions, indicating that the major feature extraction component does not introduce additional parameters during incremental learning. The session-specific parameters are much smaller than the shared backbone, and only a lightweight component is added for each incremental session. This demonstrates that the proposed parameter-isolation design can preserve session-specific knowledge while keeping the additional storage cost relatively limited. Therefore, the method achieves a favorable balance between mitigating catastrophic forgetting and maintaining parameter efficiency.

### 5.5. Ablation Study

To assess the efficacy of our methods, we conduct ablation experiments on CIFAR-100. The detailed results are provided in Table 3. After applying the forward-compatible framework, accuracy in the base session rises to 82.93%. This suggests that the method effectively enhances backbone feature extraction. However, deploying parameter isolation with model selection yielded unsatisfactory performance due to category imbalance in UQ. We address this with the SR strategy during testing, resulting in AA increasing from 58.38% to 65.27%. This underscores the importance of addressing category imbalance in UQ within the FSCIL domain. Furthermore, fine-tuning the model with real categories after forward compatibility increased AA by 2.17%. In summary, our method raised the last session accuracy from 49.04% to 54.73% and AA from 62.88% to 67.44%. This indicates that our method effectively resists forgetting and generalizes to new categories.

## 6. Conclusions

This paper proposes an FSCIL method inspired by cortical memory organization. This is the first parameter-isolation method in FSCIL. We train distinct classification models for each session to uphold the performance of the entire classification system on old categories. During testing, we quantify the uncertainty of the samples in deriving session labels, facilitating the selection of the appropriate classification model. By drawing on the organized and distributed nature of cortical memory, our approach renders the learning process of FSCIL more aligned with biologically inspired memory organization, offering a novel perspective to the field of FSCIL. In the future, we aim to develop UQ methods that are better suited for limited samples and meticulously consider detailed feature differences. Despite its effectiveness, the proposed method still requires extra storage for session-specific components, which may become costly when many incremental sessions are involved. Its entropy-based model selection also relies on reliable uncertainty estimates, and poorly calibrated session models may affect the selection accuracy. In future work, we will explore more compact parameter-sharing mechanisms, more diverse uncertainty quantification techniques that can provide robust and well-calibrated confidence estimates under limited-sample conditions, and adaptive model-selection strategies. We will also evaluate the framework on larger-scale, cross-domain, and open-world scenarios to further verify its robustness and practical applicability.

## Figures and Tables

**Table 1:** The accuracy of each session among the compared methods on CIFAR-100. AA is average accuracy.

Method	Accuracy in Each Session (%)									AA
	0	1	2	3	4	5	6	7	8	
EEIL [40]	64.10	53.11	43.71	35.15	28.96	24.98	21.01	17.26	15.85	33.79
LUCIR [23]	64.10	53.05	43.96	36.97	31.61	26.73	21.23	16.78	13.54	34.21
TOPIC [26]	64.10	55.88	47.07	45.16	40.11	36.38	33.96	31.55	29.37	42.62
CEC [25]	73.07	68.88	65.26	61.19	58.09	55.57	53.22	51.34	49.14	59.52
F2M [28]	71.45	68.10	64.43	60.80	57.76	55.26	53.53	51.57	49.35	59.13
MetaFSCIL [29]	74.50	70.10	66.84	62.77	59.48	56.52	54.36	52.56	49.47	60.73
Entropy-reg [30]	74.40	70.20	66.54	62.51	59.71	56.58	54.52	52.39	50.14	60.77
GKEAL [31]	74.01	70.47	67.01	63.08	60.01	57.30	55.50	53.39	51.40	61.35
Ours	85.30	79.94	74.16	69.19	65.40	62.09	59.40	57.16	54.73	67.44

**Table 2:** Parameter statistics of the proposed model across different sessions. The specific values will be filled in after parameter counting.

Model Component	Session								
	0	1	2	3	4	5	6	7	8
Shared backbone	11.16 M	11.16 M	11.16 M	11.16 M	11.16 M	11.16 M	11.16 M	11.16 M	11.16 M
Session-specific	30.72 K	2.56 K	2.56 K	2.56 K	2.56 K	2.56 K	2.56 K	2.56 K	2.56 K

**Table 3:** The comprehensive ablation experiment on CIFAR-100. FCis the forward-compatible framework. DR is decoupling in representation learning. MS is the model selection based on UQ. SR is the sub-results of the classification model in the base session. FT is the fine-tuning for the number of category classifiers.

FC	DR	MS	SR	FT	Accuracy in Each Session (%)									AA
					0	1	2	3	4	5	6	7	8	
					80.92	75.86	69.81	65.45	61.13	57.39	54.41	51.99	49.04	62.88
✓					82.93	76.35	71.22	66.32	61.88	58.65	55.71	52.71	49.37	63.90
✓	✓	✓			82.93	72.06	64.45	59.03	55.51	51.49	49.08	46.95	43.98	58.38
✓	✓	✓	✓		82.93	77.60	71.90	67.02	63.62	59.92	56.94	54.73	51.79	65.27
✓	✓	✓	✓	✓	85.30	79.94	74.16	69.19	65.40	62.09	59.40	57.16	54.73	67.44

## Data Availability

The publicly available datasets used in this study are presented in the manuscript.
